# Supplementary material for: Identification of photoperiod-induced specific miRNAs in the adrenal glands of Sunite sheep (Ovis aries)
Source: Front Vet Sci. 2022 Jul 22;9:888207. doi: 10.3389/fvets.2022.888207 (PMC9354845; doi:10.3389/fvets.2022.888207)
Supplement: Supplementary file 1 [file Data_Sheet_1.ZIP › Supplementary file/miRDeep2 file.zip!/miRDeep2 file/Know_Structure/SP42A1/oar-mir-495.pdf]

miRBase precursor : oar-mir-495
Total read count : 10619
oar-miR-495-5p read count : 52
oar-miR-495-3p read count : 10560
remaining reads : 7

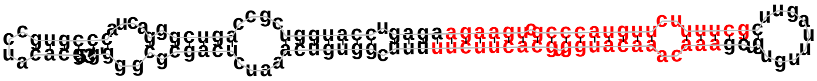

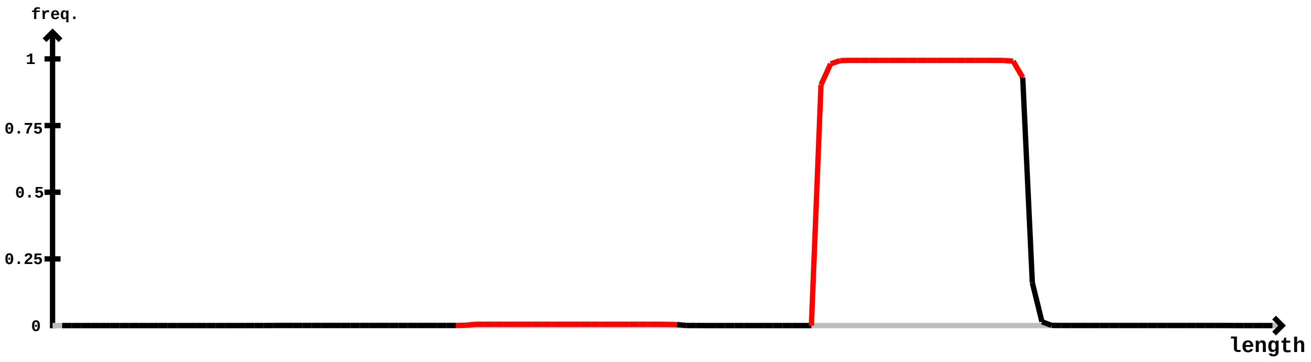


```
                                                                                oar-miR-495-3p
                                             oar-miR-495-5p
5'- gcacauccgugcccaucagggcugaccgcugguaccugagaagaagucgcccauguucuuuucgcuugauuugugucgaaacaaacauggugcacuucuuuuucggugucaaaucucagcgcgggg  -3'  exp
    ((((....))))((....(.(((((....(((((((.((((((((((.(((((((((..((((((.........).)))))..))))))).))))))))))))).)))))))....))))).)..))  reads  mm
    ..................gggcugaccgcugguaccugagaa....................................................................................  1  0
    .....................cugaccgcugguaccugagaa....................................................................................  2  0
    ......................ugaccgcugguaccugagaa....................................................................................  1  0
    .........................................agaagucgcccauguucuuuuc...............................................................  3  0
    .........................................agaagucgcccauguucuuuucg..............................................................  4  0
    .........................................agaagucgcccauguucuuuucgcu............................................................  1  0
    ..........................................gaagucgcccauguucuuuu................................................................  3  0
    ..........................................gaagucgcccauguucuuuuc...............................................................  6  0
    ..........................................gaagucgcccauguucuuuucg..............................................................  34  0
    ..........................................gaagucgcccauguucuuuucgcu............................................................  1  0
    ..............................................................................aaacaaacauggugcacu..............................  1  0
    ..............................................................................aaacaaacauggugcacuu.............................  2  0
    ..............................................................................aaacaaacauggugcacuuc............................  24  0
    ..............................................................................aaacaaacauggugcacuucu...........................  607  0
    ..............................................................................aaacaaacauggugcacuucuu..........................  7799  0
    ..............................................................................aaacaaacauggugcacuucuuu.........................  1111  0
    ..............................................................................aaacaaacauggugcacuucuuuu........................  42  0
    ...............................................................................aacaaacauggugcacuuc............................  3  0
    ...............................................................................aacaaacauggugcacuucu...........................  44  0
    ...............................................................................aacaaacauggugcacuucuu..........................  360  0
    ...............................................................................aacaaacauggugcacuucuuu.........................  402  0
    ...............................................................................aacaaacauggugcacuucuuuu........................  25  0
    ................................................................................acaaacauggugcacuucu...........................  2  0
    ................................................................................acaaacauggugcacuucuu..........................  14  0
    ................................................................................acaaacauggugcacuucuuu.........................  39  0
    ................................................................................acaaacauggugcacuucuuuu........................  76  0
    .................................................................................caaacauggugcacuucuuu.........................  1  0
    .................................................................................caaacauggugcacuucuuuu........................  5  0
    .................................................................................caaacauggugcacuucuuuuu.......................  3  0
    ....................................................................................................uuucggugucaaaucucagcg.....  2  0
    ....................................................................................................uuucggugucaaaucucagcgc....  1  0
```
